# Rights‐Based Involvement of People With Lived Experience in Pre‐Registration Occupational Therapy Education: A Scoping Review Protocol

**DOI:** 10.1111/hex.70809

**Published:** 2026-08-21

**Authors:** Ciara Ryan, Juman Simaan, Fiona Maclean

**Affiliations:** ^1^ School of Health and Social Care Edinburgh Napier University Edinburgh UK

**Keywords:** co‐production, occupational therapy education, patient and public involvement, PPIE, rights‐based approaches, service user involvement

## Abstract

**Background:**

Patient and public involvement and engagement (PPIE) is increasingly prioritised in health professional education; however, how it is conceptualised, implemented, and evaluated within pre‐registration occupational therapy programmes remains unclear. This protocol outlines a scoping review designed to map how PPIE in occupational therapy education is conceptualised, operationalised, and reported internationally, and to identify reported barriers, facilitators, and outcomes.

**Methods:**

This scoping review will adhere to the methodological framework of Arksey and O'Malley (2005), as refined by Levac et al. (2010). We will search seven databases (MEDLINE, CINAHL, PsycINFO, ERIC, ASSIA, Web of Science and OTseeker) and grey literature from 2000 onward. Using the Person‐Concept‐Context framework, eligible sources will include peer‐reviewed empirical studies and recognised grey literature reporting PPIE in pre‐registration occupational therapy education. Two reviewers will independently screen titles/abstracts and full texts, and extract data using a piloted charting tool, with third‐reviewer resolution. Findings will be synthesised using descriptive statistics and directed content analysis through a rights‐based lens. Reporting will follow PRISMA‐ScR.

**Discussion:**

The review will generate the first occupational therapy specific, rights‐based synthesis of how PPIE is conceptualised, enacted, and evaluated in pre‐registration education internationally. Findings will inform evidence‐based, feasible, and equitable approaches to involvement, offering co‐created recommendations for educators, programme leaders, and regulatory bodies. This work will also identify gaps to guide future research and policy.

**Lived Experience or Public Contribution:**

People with lived experience who contribute to occupational therapy education will be recruited as contributors to this review and involved at key stages, including search strategy development, screening, data interpretation, and synthesis. Contributors will be supported and remunerated in accordance with National Institute for Health and Care Research (2024) and Royal College of Occupational Therapists (2022) guidance, and their involvement will be documented using the GRIPP2 (Staniszewska et al., 2017) reporting framework.

**Abbreviations**
AHP: Allied Health Professions
ASSIA: Applied Social Sciences Index and Abstracts
ERIC: Education Resources Information Center
GRIPP2: Guidance for Reporting Involvement of Patients and the Public 2
HCPC: Health and Care Professions Council
MeSH: Medical Subject Headings
OSF: Open Science Framework
PCC: Population, Concept, Context
PPIE: Patient and Public Involvement and Engagement
PRESS: Peer Review of Electronic Search Strategies
PRISMA‐ScR: Preferred Reporting Items for Systematic Reviews and Meta‐Analyses Extension for Scoping Reviews
RCOT: Royal College of Occupational Therapists
WFOT: World Federation of Occupational Therapists

## 1 Introduction

Healthcare education has increasingly shifted from provider‐centric profession‐centred models toward approaches that position patients, service users, carers, and communities as partners throughout professional learning and development [1, 2]. This evolution reflects increasing acknowledgement that educational quality, person‐centredness, and social accountability are strengthened when people with lived experience contribute meaningfully to professional learning [3]. Within this broader landscape, occupational therapy holds a distinctive commitment to rights‐based and collaborative practice, making involvement particularly integral to its educational ethos [4, 5].

Patient and public involvement and engagement (PPIE) is commonly used as an umbrella term encompassing consultation, collaboration, and co‐production, through which people with lived experience meaningfully influence decision‐making processes in the design, delivery, evaluation, and governance of programmes or research [6]. In this review, involvement is explicitly conceptualised through a rights‐based lens that recognises people accessing occupational therapy services as rights‐bearers whose autonomy, experiential knowledge, and participation are foundational to occupational and human rights [7]. Rights‐based approaches emphasise that equitable and justice‐oriented health professional education must be shaped with, rather than for, the individuals and communities it intends to serve [8]. Related terms such as service user involvement, consumer participation, and co‐production occupy different positions along an engagement continuum [9]. For clarity and consistency, this review uses the term rights‐bearer involvement while acknowledging this broader terminology.

The evidence base for PPIE in health professional education is expanding rapidly, and three consistent patterns emerge across recent syntheses. First, the benefits of involvement are well established: a Best Evidence Medical Education systematic review identified that meaningful involvement enhances learners' empathy, communication skills, and readiness for person‐centred care [10], and an umbrella review synthesising 20 reviews reported substantial and consistent evidence that public participation in healthcare students' education enhances outcomes for learners, the public, curricula, and healthcare systems [11]. Second, the evidence is unevenly distributed across the professions, originating predominantly from medicine and nursing [11, 12]. Third, where the allied health professions are examined, active involvement remains largely confined to teaching delivery at the lower levels of Towle et al.'s (2010) taxonomy, with minimal participation in curriculum design or governance and no evaluation of long‐term clinical outcomes [13]. Furthermore, a review by Shand et al., [13] was intentionally focused on active forms of involvement within undergraduate programmes and did not seek to map involvement across the wider range of educational activities and programme routes that characterise contemporary occupational therapy education. Taken together, these syntheses demonstrate both the value of involvement and a persistent gap: none provides a discipline‐specific account of how involvement is conceptualised, enacted, and evaluated within pre‐registration occupational therapy education, underscoring the need for further systematic investigation of this topic.

Internationally, the World Federation of Occupational Therapists' Minimum Standards for the Education of Occupational Therapists [14] explicitly require programmes to collaborate with service users in curriculum development, programme evaluation, and educational methods, aligning with global expectations for inclusive, participatory, and socially responsive occupational therapy education. Within the United Kingdom, the regulatory framework has progressively strengthened the expectations placed on approved education providers. The Health and Care Professions Council's (HCPC) Standards of Education and Training (2017) require meaningful service user and carer involvement across all aspects of programme design and delivery. A HCPC‐commissioned study examining service user involvement across 540 approved programmes found that although 71% reported some form of involvement, the depth, consistency, and sustainability of this activity varied widely, with most participation concentrated in programme development rather than more influential partnership roles [15]. Most recently, the Royal College of Occupational Therapists' (RCOT) revised Learning and Development Standards for Pre‐Registration Education [16] require that people who access occupational therapy services are meaningfully involved in the development, enhancement, and delivery of programmes, including admissions, curriculum design and delivery, practice‐based learning, and ongoing programme review (Standard 3.20), extending this expectation across the full educational lifecycle. At a professional level, RCOT's Research and Innovation Strategy 2025–2035 [17] identifies knowledge co‐creation and meaningful partnership with people accessing services as central professional priorities. The James Lind Alliance Priority Setting Partnership further confirmed that strengthening education as a vehicle for service improvement is a key professional ambition shared by practitioners, researchers, and people using services alike [18].

Despite longstanding professional commitments, no existing synthesis examines how rights‐bearer involvement is understood, operationalised, and evaluated across the breadth of pre‐registration occupational therapy education internationally. This gap is evident from a systematic comparison of the scope of existing reviews: syntheses to date have addressed medical education [10], nursing education [12], interprofessional education [19], the allied health professions collectively [13], and healthcare students' education broadly [11], while the only occupational therapy‐specific scoping reviews of involvement to date focused on health research rather than pre‐registration education [20, 21]. The review closest in scope, by Shand et al. [13], in fact strengthens rather than diminishes the case for the present work. Occupational therapy was the most represented profession in that review, accounting for 13 of its 25 included studies, yet, as an allied health‐wide synthesis, it did not analyse or report occupational therapy findings at discipline level. Its eligibility criteria were also deliberately narrower than those proposed here: it was restricted to active involvement (levels 3–6 of Towle et al.'s (2010) taxonomy) within undergraduate programmes, and it excluded admissions processes, practice‐based learning without an academic component, and simulated learning. By design, therefore, it cannot map consultation‐level involvement, pre‐registration master's and apprenticeship routes, or involvement across admissions, practice‐based learning, assessment, and programme governance, precisely the full educational lifecycle across which United Kingdom regulatory and professional standards now mandate involvement [16, 22].

Occupational therapy education providers are consequently in the position of being required to implement involvement across the full educational lifecycle while lacking any synthesis of how such involvement is currently conceptualised, enacted, or evaluated. As a result, important questions remain regarding how involvement is enacted across different educational contexts, how occupational therapy's client‐centred and rights‐based values are reflected in educational practice, and how outcomes for students, contributors, and institutions are assessed and reported. By systematically mapping the nature, scope, and characteristics of involvement activities, together with their reported rationales, outcomes, barriers, and facilitators, this review aims to provide the first occupation‐specific synthesis of involvement in pre‐registration occupational therapy education and to inform future educational development, research, and policy.

## 2 Methods

### 2.1 Design

This scoping review will follow the methodological framework developed by Arksey and O'Malley [23], enhanced by Levac et al.'s (2010) recommendations. Reporting will follow the PRISMA‐ScR checklist [24]. The protocol is registered with the Open Science Framework (DOI: 10.17605/OSF. IO/QZXG5).

A critical realist epistemology [25] underpins the review, recognising that rights‐bearer involvement practices exist within complex, context‐dependent systems while maintaining that meaningful patterns and transferable insights can be identified through systematic investigation. This approach acknowledges the socially constructed nature of involvement while maintaining commitment to rigorous evidence synthesis that can inform practice improvement.

### 2.2 Stage 1: Identifying the Research Question

The overarching question guiding this scoping review is: How is rights‐bearer involvement and engagement implemented, rationalised, and evaluated in pre‐registration occupational therapy education internationally? Consistent with scoping review methodology, which is intended to map the breadth and nature of evidence in a field [23, 26] this deliberately broad question is addressed through four clearly delineated sub‐questions, each mapped to a discrete domain of the data‐charting framework:

1. What theoretical and practical rationales are reported for rights‐bearer involvement in pre‐registration occupational therapy education?
2. What processes, methods, and approaches are used to implement involvement across admissions, curriculum design, teaching, assessment, and programme governance?
3. How is involvement evaluated, including the outcomes measured across stakeholder groups and the frameworks or tools used to assess effectiveness?
4. What barriers, facilitators, and contextual factors shape the implementation of involvement initiatives?

Structuring the review in this way retains the breadth appropriate to scoping methodology while ensuring that rationales, implementation, evaluation, and context are examined and reported as distinct elements rather than conflated.

### 2.3 Stage 2: Identifying Relevant Studies

A comprehensive search strategy will be developed in collaboration with experienced health sciences and education librarians. This strategy will draw on established patient and public involvement search filters and adapt them specifically for occupational therapy education contexts [27]. To ensure full reproducibility, a complete draft search strategy for MEDLINE (Ovid) is provided in Appendix 1. Prior to execution, the search strategies will be peer reviewed using the Peer Review of Electronic Search Strategies (PRESS) checklist [28, 29], and will be translated for the syntax, controlled vocabulary, and field codes of each remaining database. The final search strategies for all databases, together with the dates of searching, any amendments made following PRESS review, and the number of records retrieved, will be reported in full as supporting material to the completed review.

Seven electronic databases will be searched from 2000 onwards to ensure comprehensive coverage across health, education, occupational therapy, and social science literature: MEDLINE, CINAHL, PsycINFO, ERIC, ASSIA, Web of Science and OTseeker. The search strategy will combine three concept clusters using Boolean operators, with terms developed through iterative testing and refinement (see Table 1). Recognising that ‘rights‐based’ terminology is not consistently used within the literature, search terms prioritise established PPIE terminology, with the rights‐based lens applied analytically during synthesis. Controlled vocabulary terms (MeSH, CINAHL headings) will be supplemented with comprehensive free‐text searching to capture emerging terminology and international language variations.

**Table 1:** Overview of search concepts and example terms.

Concept	Search Terms
‘Rights‐bearer’ and PPIE terms	“Patient and public involvement,” “service user involvement,” “consumer participation,” “client engagement,” “co‐production,” “participatory,” “collaborative partnership,” “stakeholder engagement,” and “lived experience.” (with reference to the established PPIE search filter developed by [27])
	“Rights‐bearer,” “rights‐based involvement,” or “occupational rights”
AND	
Occupational therapy terms	“Occupational therapy,” “occupational therapist,” “occupational science,” and related terms.
AND	
Education terms	“Education,” “curriculum,” “teaching,” “learning,” “student,” “pre‐registration,” “clinical education,” and “fieldwork.”

Hand‐searching reference lists of all included studies and forward citation searching will be conducted. Grey literature will be systematically searched across multiple sources, including Google Scholar, OpenGrey, ProQuest Dissertations and Theses Global, websites and repositories of professional occupational therapy organisations (e.g., the Royal College of Occupational Therapists, the American Occupational Therapy Association, the World Federation of Occupational Therapists, and the Council of Occupational Therapists for the European Countries), national occupational therapy associations, reports from healthcare education regulators, major international conference proceedings, university and higher‐education research repositories, and specialist public‐involvement networks such as UK public‐involvement guidance bodies (e.g., INVOLVE).

Consistent with methodological guidance, grey literature searching will adopt a pragmatic yet transparent approach, recognising the lack of standardised methods and the resource‐intensive nature of identifying and screening such evidence. Google Scholar will be used as a supporting search tool rather than a primary database, and screening will be limited to the first 200 results for each search string, reflecting evidence that relevant studies, including grey literature, frequently occur beyond early search pages and that screening up to 200–300 results improves retrieval sensitivity [30]. All limits applied to search outputs (e.g., number of results screened) will be explicitly reported to enhance transparency and reproducibility, in line with recommendations that grey literature search parameters should be clearly documented [31].

### 2.4 Stage 3: Study Selection

All retrieved records will be imported into EndNote for systematic de‐duplication, after which the de‐duplicated library will be uploaded into Rayyan for title/abstract and full‐text screening and for documenting screening decisions.

Study selection will then follow a two‐phase screening process to ensure systematic, transparent, and replicable identification of relevant studies. In the first phase, two reviewers will independently screen all titles and abstracts against the predefined inclusion criteria. Any discrepancies will be resolved through discussion, with a third reviewer available to adjudicate when consensus cannot be reached. In the second phase, full text articles of all potentially eligible studies will be retrieved and independently assessed by the same reviewers. A standardised form will be used to document reasons for exclusion at the full text stage. The full selection process will be reported using the PRISMA‐ScR flow diagram [24], ensuring transparency and replicability of the review methods.

Studies will be included if they involve any level of rights‐bearer involvement in pre‐registration occupational therapy education, from minimal consultation to full co‐production models. We will exclude studies that only describe rights‐bearer involvement without any evidence of actual implementation or engagement activities. Full eligibility criteria are outlined in Table 2.

**Table 2:** Eligibility criteria (PCC framework).

PCC domain	Inclusion criteria	Exclusion criteria
Population	Studies involving people with lived experience, service users, carers, or rights‐bearer contributors participating in any aspect of *pre‐registration occupational therapy education*.	Studies involving only students, educators, practitioners, or researchers without any involvement of people with lived experience.
Concept	Empirical studies or formal programme evaluations reporting implemented PPIE activities (e.g., consultation, collaboration, partnership, or co‐production).	Studies describing *proposed* PPIE activities with no implementation; papers that mention involvement superficially without describing specific activities.
Context	Pre‐registration occupational therapy education internationally, including university‐based teaching, online learning, simulation, practice‐based learning, admissions processes, curriculum codesign, or programme governance.	Post‐registration or continuing professional development; studies focused solely on clinical practice without an educational component; studies unrelated to education.
Source Type	Peer‐reviewed empirical studies of any design; grey literature from recognised professional or educational bodies; publications in English from 2000 onwards.	Conference abstracts, commentaries, editorials, opinion pieces (unless they report formal programme evaluation); non‐English publications; studies published before 2000.

### 2.5 Stage 4: Charting the Data

A data‐charting form will be developed iteratively (see supporting information file I) independently piloted by two reviewers on three included studies before full extraction. Data extracted will include publication characteristics; the educational setting and nature of the involvement activity; roles, contributions, and preparatory processes for contributors; theoretical or pedagogical frameworks informing the involvement; reported outcomes for students, staff, institutions, or contributors; evaluation approaches; and identified barriers and facilitators.

Two reviewers will independently extract data to enhance consistency. The charting process will remain flexible, enabling refinement of categories as familiarity with the literature develops [23].

### 2.6 Stage 5: Collating, Summarising, and Reporting the Results

The analysis will employ both quantitative and qualitative approaches to provide a comprehensive understanding of the evidence.

Quantitative elements (e.g., publication year, involvement types, countries of origin) will be summarised descriptively. Qualitative data will undergo directed content analysis informed by the research questions [32]. Findings will be synthesised into themes that describe how involvement is rationalised, enacted, supported, and evaluated across international occupational therapy education.

Results will be presented through a combination of narrative synthesis, summary tables, and visual mapping of involvement activities across educational contexts. Gaps in the evidence base will be identified to inform future research, policy development, and curriculum design.

### 2.7 Stage 6: Stakeholder Consultation

In line with Levac et al.‘s (2010) recommendation that consultation forms an essential knowledge‐generating stage of scoping review methodology, people with lived experience who currently contribute to occupational therapy education will be recruited as contributors to this review. Consistent with the rights‐based framing underpinning this work, lived experience contributors will not be engaged as passive consultees at a single endpoint, but as active partners involved across multiple stages of the review process.

Contributors will be recruited purposively to reflect diversity in terms of the nature of their lived experience and their current roles within occupational therapy education. Involvement will span key stages of the review, including input into screening processes (e.g., refining eligibility criteria, resolving uncertainties where appropriate), support with screening decisions where appropriate, engagement with emerging themes during data interpretation, and contribution to the development of rights‐aligned recommendations during synthesis. Contributors will be supported and remunerated in accordance with National Institute for Health and Care Research (NIHR) [33] and Royal College of Occupational Therapists [34] guidance, and their involvement will be documented using the GRIPP2 [35] reporting framework. Contributors will be invited to co‐author the final review paper, reflecting best practice in equitable and collaborative research dissemination.

Ethical approval for the involvement of lived experience contributors will be sought from the School of Health and Social Care Ethics Committee at Edinburgh Napier University.

## 3 Methodological Rigour

Alongside transparent documentation, a series of methodological safeguards will be embedded to minimise bias throughout the review. The search strategy will be peer reviewed by an information specialist using the PRESS checklist [28] to reduce the risk of retrieval bias. Before formal screening begins, the review team will complete a calibration exercise on a random sample of records to establish a shared understanding of the eligibility criteria; screening and data extraction will then be undertaken independently by two reviewers, with disagreements resolved through discussion and third‐reviewer adjudication, thereby reducing the risk of selection and extraction bias. Consistent with established scoping review guidance, critical appraisal of included sources will not be undertaken, as the purpose of the review is to map the extent and nature of the evidence rather than to evaluate its quality (Peters et al., 2021); this decision, and its implications for interpreting the findings, will be stated transparently in the completed review. Finally, reflexivity will be actively addressed: as the authors are occupational therapy educators with a stated commitment to involvement, emerging interpretations will be tested through team discussion and through consultation with lived experience contributors, providing an interpretive check on researcher assumptions.

A comprehensive audit trail will be maintained throughout the review process. A detailed methodological log will document the development and implementation of all review processes, including search strategy development, screening decisions, data extraction procedures, analysis methods, and synthesis approaches. This log will be consistently updated during active phases of the review, with regular quality checks to ensure completeness and accuracy.

Decision‐making documentation will follow a standard format capturing the context and background of each decision, options considered, supporting evidence, implementation steps, and review mechanisms. All documentation will be cross‐referenced to relevant project documents, with regular audits ensuring completeness of records.

Changes to the protocol will be recorded in a formal change log that details the date and nature of each modification, rationale for change, impact assessment, approval process, and implementation plan. A version control system will track all document revisions, with regular reviews assessing the impact of any changes on the overall project.

### 3.1 Limitations

Potential limitations include inconsistent terminology across international contexts, variability in occupational therapy programme structures, and the restriction to English language sources. Variations in reporting quality may limit comparability. The iterative nature of scoping review methodology and the ongoing involvement of lived experience contributors across review stages will support the mitigation of these challenges.

## 4 Discussion

Following the established methodological guidance for scoping reviews [23, 36] outlined in the method section above and based on our reading of the literature we reviewed during the writing of this protocol, this section outlines a possible synthesis of the anticipated findings of this proposed review, and how they might relate to research and practice in the area.

This scoping review is designed to provide a comprehensive, rights‐based synthesis of how people with lived experience are involved in pre‐registration occupational therapy education internationally. By mapping the breadth of involvement activities, underpinning rationales, and reported outcomes, the review seeks to clarify how PPIE is currently conceptualised and enacted across diverse educational contexts. Such insight is essential given longstanding calls for more meaningful and equitable partnership working in health professional education, and ongoing concerns regarding tokenistic or inconsistent involvement [3].

A key anticipated contribution of this review is its explicit engagement with rights‐based perspectives. Occupational therapy has a distinctive professional commitment to occupational and human rights, yet it remains unclear how these principles translate into pedagogical practice within pre‐registration programmes. Synthesising evidence through a rights‐bearer lens will help highlight the extent to which involvement is treated not simply as an educational enhancement, but as an ethical and epistemic imperative grounded in justice, autonomy, and shared decision‐making.

The review is expected to shed light on the considerable conceptual and terminological variation that characterises the involvement literature [5]. Understanding how different programmes describe and operationalise partnership, whether as service user involvement, co‐production, client engagement, or PPIE, will help identify both shared principles and areas where conceptual ambiguity may impede evaluation, comparison, or implementation. By documenting the range of outcomes measured, as well as the methods and frameworks used to assess them, the review may also highlight the need for stronger, more consistent evaluation tools that capture impacts for all stakeholder groups [35], including rights‐bearer contributors.

Finally, the sustained involvement of lived experience contributors across multiple review stages is intended to ensure that findings will be interpreted through the priorities, expertise, and lived realities of those most directly affected by involvement practices. Rather than treating lived experience as a consultative endpoint, this approach positions contributors as partners throughout the scoping review process, which is expected to enhance the relevance, accuracy, and practical value of the review. The co‐produced recommendations emerging from this collaboration are intended to support educators and institutions to implement involvement that is meaningful, sustainable, and grounded in rights‐based principles. At a broader level, public participation in healthcare education has been shown to generate benefits across learner, curriculum, and system‐level outcomes, while also highlighting ongoing challenges in implementation and sustainability [11]. Collectively, the review aims to provide a robust evidence base to inform future curriculum design, regulatory guidance, and research in occupational therapy.

## Author Contributions

**Ciara Ryan:** conceptualisation, writing – original draft, methodology, writing – review and editing, investigation. **Juman Simaan:** writing – review and editing, methodology, conceptualisation. **Fiona Maclean:** writing – review and editing, conceptualisation, methodology.

## Ethics Statement

Ethical approval for the involvement of people with lived experience as contributors to this review will be sought from the School of Health and Social Care Ethics Committee, Edinburgh Napier University.

## Conflicts of Interest

The authors declare no conflicts of interest.

## Supporting information


Supporting File

## Data Availability

The data that support the findings of this study are available from the corresponding author upon reasonable request.
