# Supplementary material for: Rights‐Based Involvement of People With Lived Experience in Pre‐Registration Occupational Therapy Education: A Scoping Review Protocol
Source: Health Expect. 2026 Aug 21;29(4):e70809. doi: 10.1111/hex.70809 (PMC13494670; doi:10.1111/hex.70809)
Supplement: Supplementary file 1 [file HEX-29-e70809-s001.docx]

**Supplementary material**

**Appendix 1: Draft search strategy for MEDLINE (Ovid)**

| **Line** | **Search statement** |
| --- | --- |
| 1 | exp Patient Participation/ |
| 2 | exp Community Participation/ or exp Stakeholder Participation/ |
| 3 | (“patient and public involvement” or “public involvement” or “patient involvement” or PPI or PPIE).ti,ab. |
| 4 | (“service user involvement” or “service user participation” or “consumer participation” or “consumer engagement” or “client engagement” or “client participation”).ti,ab. |
| 5 | (co-production or coproduction or co-design or codesign or co-creation or participatory or “collaborative partnership*” or “stakeholder engagement”).ti,ab. |
| 6 | (“lived experience*” or “expert* by experience” or “rights-bearer*” or “rights-based involvement” or “occupational rights”).ti,ab. |
| 7 | or/1-6 |
| 8 | exp Occupational Therapy/ |
| 9 | (occupational therap* or “occupational science”).ti,ab. |
| 10 | or/8-9 |
| 11 | exp Education, Professional/ or exp Curriculum/ or exp Students, Health Occupations/ |
| 12 | (education* or curricul* or teaching or learning or student* or pre-registration or preregistration or undergraduate or “clinical education” or fieldwork or “practice placement*”).ti,ab. |
| 13 | or/11-12 |
| 14 | 7 and 10 and 13 |
| 15 | limit 14 to yr=”2000-Current” |

**Appendix 2: Data extraction form**

| **Domain** | **Field** | **Data to be extracted** |
| --- | --- | --- |
| **Study Characteristics** | Study identification | Authors, year of publication, country |
|  | Source type | Peer-reviewed article, grey literature, thesis or report; funding source (if reported) |
|  | Study design and methodology | Study design, methodological approach, and theoretical orientation (if stated) |
|  | Aims and frameworks | Study aims, research questions, and any conceptual or theoretical frameworks used |
| **Educational Context** | Setting and programme | Type of institution; programme level (e.g. undergraduate, postgraduate); geographical context |
|  | Curriculum context | Area of the programme where involvement occurs (e.g. teaching, placement, assessment, admissions, governance) |
|  | Participants | Description of students, educators, and contributors with lived experience (where reported) |
|  | Organisational context | Relevant institutional or regulatory factors influencing implementation (if reported) |
| **Involvement of ‘Right-Bearers’** | Terminology and definitions | Terms used to describe involvement and any definitions or conceptual models reported |
|  | Contributor characteristics | Description of contributors with lived experience, including recruitment and selection processes |
|  | Nature of involvement | Level, timing, and scope of involvement within the educational programme |
|  | Activities | Types of involvement (e.g. teaching, curriculum development, assessment, governance) |
|  | Preparation and support | Training, support mechanisms, and preparation for contributors and staff (if reported) |
|  | Resources | Financial, staffing, or infrastructural resources required (if reported) |
| **Implementation** | Barriers | Reported challenges to implementing involvement |
|  | Facilitators | Factors that supported implementation |
|  | Contextual factors | Broader influences such as organisational culture, power relations, or sustainability considerations (if reported) |
|  | Adaptations | Reported changes or refinements to involvement approaches over time |
| **Outcomes and Impacts** | Student outcomes | Reported effects on student knowledge, skills, attitudes, or development |
|  | Staff or institutional outcomes | Effects on teaching practices, curriculum design, or organisational processes |
|  | Contributor outcomes | Reported impacts on people with lived experience involved (if described) |
|  | Programme or system outcomes | Wider reported impacts (e.g. accreditation, policy influence, community engagement) |
| **Evaluation** | Evaluation approach | Methods used to assess involvement (qualitative, quantitative, mixed methods) |
|  | Measures and data sources | Types of data collected (e.g. surveys, interviews, observations) |
|  | Limitations | Reported limitations of evaluation or study design |
| **Additional Information** | Key points | Any additional relevant findings not captured above |
|  | Recommendations | Author-reported implications for practice, policy, or research |
